# Wearable biofeedback device to assess gait features and improve gait pattern in people with parkinson’s disease: a case series

**DOI:** 10.1186/s12984-024-01403-z

**Published:** 2024-06-26

**Authors:** Thomas Bowman, Andrea Pergolini, Maria Chiara Carrozza, Tiziana Lencioni, Alberto Marzegan, Mario Meloni, Nicola Vitiello, Simona Crea, Davide Cattaneo

**Affiliations:** 1 IRCCS Fondazione Don Carlo Gnocchi, Milan, Italy; 2 The BioRobotics Institute, Scuola Superiore Sant’Anna, Pisa, 56127 Italy; 3 Department of Excellence in Robotics & AI, Scuola Superiore Sant’Anna, Pisa, 56127 Italy; 4 Department of Physiopathology and Transplants, University of Milan, Milan, Italy; 5 IRCCS Fondazione Don Carlo Gnocchi, Florence, Italy; 6 National Research Council of Italy (CNR), Rome, Italy

**Keywords:** Wearable device, Biofeedback, Parkinson’s disease, Gait parameters, Assessment, Rehabilitation

## Abstract

**Introduction:**

People with Parkinson’s Disease (PD) show abnormal gait patterns compromising their independence and quality of life. Among all gait alterations due to PD, reduced step length, increased cadence, and decreased ground-reaction force during the loading response and push-off phases are the most common. Wearable biofeedback technologies offer the possibility to provide correlated single or multi-modal stimuli associated with specific gait events or gait performance, hence promoting subjects’ awareness of their gait disturbances. Moreover, the portability and applicability in clinical and home settings for gait rehabilitation increase the efficiency in the management of PD. The Wearable Vibrotactile Bidirectional Interface (BI) is a biofeedback device designed to extract gait features in real-time and deliver a customized vibrotactile stimulus at the waist of PD subjects synchronously with specific gait phases. The aims of this study were to measure the effect of the BI on gait parameters usually compromised by the typical bradykinetic gait and to assess its usability and safety in clinical practice.

**Methods:**

In this case series, seven subjects (age: 70.4 ± 8.1 years; H&Y: 2.7 ± 0.3) used the BI and performed a test on a 10-meter walkway (10mWT) and a two-minute walk test (2MWT) as pre-training (Pre-trn) and post-training (Post-trn) assessments. Gait tests were executed in random order with (Bf) and without (No-Bf) the activation of the biofeedback stimulus. All subjects performed three training sessions of 40 min to familiarize themselves with the BI during walking activities. A descriptive analysis of gait parameters (i.e., gait speed, step length, cadence, walking distance, double-support phase) was carried out. The 2-sided Wilcoxon sign-test was used to assess differences between Bf and No-Bf assessments (*p* < 0.05).

**Results:**

After training subjects improved gait speed (Pre-trn_No-Bf: 0.72(0.59,0.72) m/sec; Post-trn_Bf: 0.95(0.69,0.98) m/sec; *p* = 0.043) and step length (Pre-trn_No-Bf: 0.87(0.81,0.96) meters; Post-trn_Bf: 1.05(0.96,1.14) meters; *p* = 0.023) using the biofeedback during the 10mWT. Similarly, subjects’ walking distance improved (Pre-trn_No-Bf: 97.5 (80.3,110.8) meters; Post-trn_Bf: 118.5(99.3,129.3) meters; *p* = 0.028) and the duration of the double-support phase decreased (Pre-trn_No-Bf: 29.7(26.8,31.7) %; Post-trn_Bf: 27.2(24.6,28.7) %; *p* = 0.018) during the 2MWT. An immediate effect of the BI was detected in cadence (Pre-trn_No-Bf: 108(103.8,116.7) step/min; Pre-trn_Bf: 101.4(96.3,111.4) step/min; *p* = 0.028) at Pre-trn, and in walking distance at Post-trn (Post-trn_No-Bf: 112.5(97.5,124.5) meters; Post-trn_Bf: 118.5(99.3,129.3) meters; *p* = 0.043). SUS scores were 77.5 in five subjects and 80.3 in two subjects. In terms of safety, all subjects completed the protocol without any adverse events.

**Conclusion:**

The BI seems to be usable and safe for PD users. Temporal gait parameters have been measured during clinical walking tests providing detailed outcomes. A short period of training with the BI suggests improvements in the gait patterns of people with PD. This research serves as preliminary support for future integration of the BI as an instrument for clinical assessment and rehabilitation in people with PD, both in hospital and remote environments.

**Trial registration:**

The study protocol was registered (DGDMF.VI/P/I.5.i.m.2/2019/1297) and approved by the General Directorate of Medical Devices and Pharmaceutical Service of the Italian Ministry of Health and by the ethics committee of the Lombardy region (Milan, Italy).

**Supplementary Information:**

The online version contains supplementary material available at 10.1186/s12984-024-01403-z.

## Introduction

People with Parkinson’s disease (PD) are characterized by a wide range of gait disturbances associated with the risk of falling, reduced independence, and low quality of life [1, 2]. The degeneration of dopaminergic and cholinergic structures affects the automatic internally-driven gait, leading to the typical bradykinetic gait characterized by reduced step length, increased cadence, and decreased plantar force during the loading response and push-off phases [3, 4]. The externally-driven circuits controlling gait are relatively preserved and can be exploited in rehabilitation to enhance a more physiological gait pattern [4].

Recent systematic reviews [5, 6] established that physical therapy interventions (e.g., treadmill training and cueing) are recommended as effective regimens for treating gait impairments in people with PD (evidence level A - according to the European Federation of Neurological Societies) [7], even if further work is required to compare the relative efficacy of the different treatments. According to the recommendations for effective gait rehabilitation, biofeedback signals can be used as an external cue or “stimuli” for improving parkinsonian locomotion, as they activate the premotor cortical system while bypassing the basal ganglia [8]. In this context, wearable devices delivering a biofeedback stimulus represent a valuable rehabilitation tool for the daily assistance of PD subjects [8, 9].

Wearable technologies offer significant opportunities in the clinical management of PD, especially considering the possibility of being used in the form of telemedicine or remote rehabilitation. Indeed, these devices could be used in a clinic to provide detailed outcomes and add needed objectivity to routine clinical tests. Moreover, the use of wearable biofeedback devices could help PD subjects with limited access to rehabilitation by enhancing their therapeutic management, offering them an approach potentially usable outside the hospital [10, 11]. Indeed, despite the demonstrated benefit of rehabilitation programs in PD, only a fraction of subjects are able to benefit from conventional physiotherapy sessions. This is due to the limited number of practices unable to satisfy the rising demand for physiotherapy, as well as to practical limits including the difficulty of organizing the service in the territory, particularly in rural and peripheral areas [12]. Finally, the COVID-19 pandemic has reinforced the urgent need for better tools to manage chronic conditions remotely, as regular access to clinics may be problematic [13]. According to previous considerations, wearable biofeedback devices for people with PD are designed to measure gait features by using wireless sensors both in supervised (i.e. hospital, clinic) and unsupervised settings (i.e. home) and to deliver a correlated single or multi-modal biofeedback stimuli (acoustic, visual, or vibratory) promoting subjects’ awareness of gait disturbances and physiological walking patterns, as indicated by recent reviews [9, 14] and pilot studies [15, 16]. In agreement with the state of the art [9], the majority of wearable biofeedback devices developed for people with PD use miniaturized wearable sensors and actuators attached to the user’s waist, head, or ankle. IMU sensors are commonly used (they are mainly placed on subjects’ waist and ankles) while pressure sensors have been used less. Winfree et al. [15]. developed a pair of shoes called “the PDShoes” embedding three pressure sensors placed at the heel, ball, and toe of the foot to provide vibrotactile stimuli. Moreover, Aggarwal et al. [16]. elucidate the effect of step-synchronized vibration using the PDShoes in a pilot study with positive effects on gait and balance outcomes.

Recently, the team of engineers of the Wearable Robotic Laboratory of the Scuola Superiore Sant’Anna developed a wearable vibrotactile Bidirectional Interface (BI) to collect healthy subjects’ gait parameters and confirm suitability for gait phase estimation and discrete event recognition [17]. Furthermore, the device has already shown potential for enhancing gait symmetry using vibrotactile biofeedback in subjects with lower-limb amputation by delivering specifically designed, short-lasting, and low-intensity vibrations synchronously with pre-defined gait events [18]. In accordance with the literature on wearable biofeedback devices in people with PD, the BI could be used to extract gait parameters during clinical tests and to provide a vibrotactile stimulus to improve their walking performance. Indeed, the current challenges in biofeedback-oriented research are directed at identifying the mechanistic factors behind the success of sustained sensory augmentation or at comparing and integrating uni- and multi-sensory stimuli [9, 19]. However, there is also an interest in identifying effective biofeedback strategies for people with PD, in particular regarding vibrotactile stimulation [5, 14]. In line with the current needs for PD, the BI could potentially become a solution for self-assessment and self-training at home or in real life context. Compared to the majority of the vibrotactile biofeedback devices tested on people with PD [14], the BI combines both IMU and pressure sensors. This configuration could potentially allow for the extraction of both kinematic and kinetic parameters in a dynamic environment (e.g. walking in the hospital corridor). In terms of self-training, a current limitation [9, 14] in wearable biofeedback devices for people with PD is the lack of a clear definition of how biofeedback should be applied in relation to different motor symptoms. The novelty of the BI is the possibility to customize the vibrotactile biofeedback stimuli and developing different biofeedback strategies according to specific gait impairments. Its application for people with PD seems suitable as a possible strategy to support personalized and intensive training, which have been proven to be two critical factors in improving rehabilitation interventions in this population [20]. Moreover, the use of vibrotactile biofeedback seems suitable for people with PD since the sensory decline of the somatosensory system seems less compromised by aging compared to hearing or vision [14, 21]. Indeed, hearing is more affected by the environment and the noise that constantly surrounds us, making it more difficult to use acoustic feedback compared to tactile somatosensory perception, especially in the external environment. Furthermore, compared to visual biofeedback, vibrotactile stimulus allows for use during task-oriented activities in a real-life context since users do not have to constantly look at a monitor or screen. According to the model proposed by Gonçalves et al. [14], vibrotactile biofeedback can be used to preserve optimal gait and balance control by exploiting the feedback with a stabilizing or augmenting role. In line with the proposed model, we designed our device to deliver a phase-dependent vibrotactile biofeedback stimulus aimed at improving gait parameters in people with PD exploiting an augmented biofeedback to increase the awareness of the impaired gait sub-phases in a semi-continuous way. These biofeedback strategies differ from those for lower-limb amputees because they have been customized according to specific gait abnormalities of people with PD.

Given its prototype nature, there are no previous studies testing the BI in people with PD. Before implementing the BI in a home setting, it is necessary to verify its effect on the gait pattern, along with its usability and safety in a clinical context with a small sample of people with PD. We hypothesize that the BI might promote an immediate effect on bradykinetic gait. Furthermore, a short training period with this biofeedback device could lead to a training effect. Therefore, to evaluate the effects, usability, and safety of the BI in subjects with PD, the main objectives of this case-series study are: (1) to assess change in gait parameters usually compromised by the typical bradykinetic gait, and (2) to report the results of the System usability scale and the number of adverse events occurred using the BI in clinical practice and as part of clinical test.

## Methods

### Wearable vibrotactile bidirectional interface

**Fig. 1:**
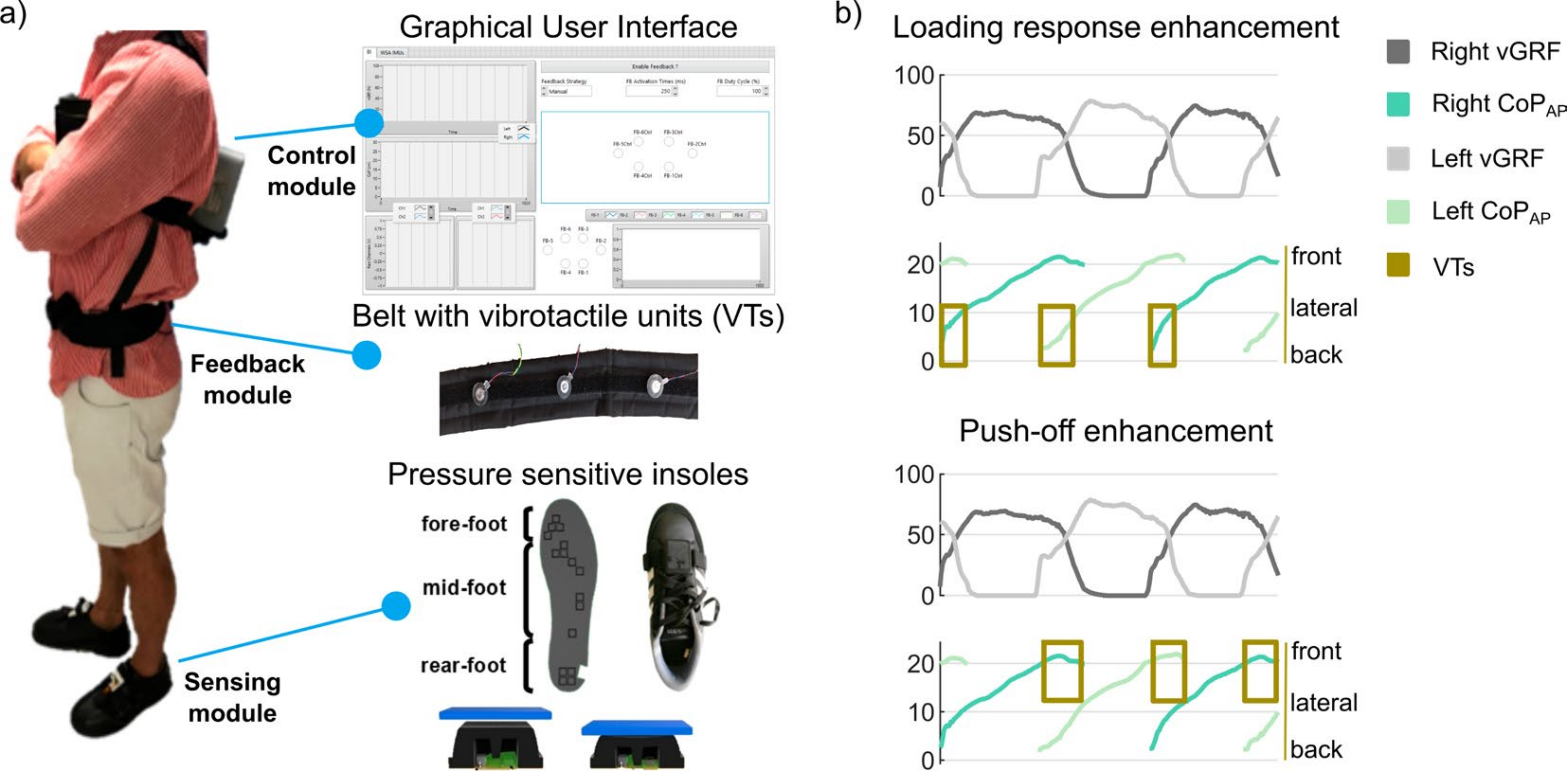
(**a**) The wearable vibrotactile Bidirectional Interface and its modules. (**b**) The two biofeedback strategies. Abbreviations: CoP, Center of pressure; vGRF, vertical ground reaction force; VTs, Vibrotactile stimulus

The Wearable Vibrotactile Bidirectional Interface, in short BI, is a fully wearable biofeedback device powered by a lithium-polymer battery (Li-ION 11.1 V) shown in Fig. 1. The BI consists of three modules: the Sensory module, the Control module, and the Feedback module.

The Sensory module is comprised of a pair of instrumented shoes with pressure-sensitive insoles equipped with 16 optoelectronic sensors, utilizing technology and patents described in [17]. Additionally, an IMU is integrated into the onboard electronics, (a microcontroller STM32L476RG, STMicroelectronics). The acquired sensor signals are wirelessly transmitted to the Control module through an embedded Ultra-Wide Band transceiver (DWM1000, DecaWave, 6.8 Mbps data rate). The BI allows for real-time measurements of the vertical ground reaction force (vGRF), the position of the center of pressure along the anteroposterior direction of the foot (CoP_AP_), the detection of gait events (i.e., heel-strike and toe-off) and the estimation of the spatiotemporal parameters of gait. Evaluating the pressure-sensitive insoles with healthy subjects in a prior study compared to gold standard (motion tracking system), the heel-strike and toe-off detection exhibited an overall median absolute error (MAE) of 0.06 s and 0.04 s for heel-strike and toe-off detection, respectively. The device additionally gives an accurate estimation of the stance phase duration with a 2.02% error [17]. This assessment was also made in a preliminary study of the bradykinetic gait of nine PD subjects, which showed a MAE of 0.028 s for heel-strike, 0.033 s for toe-off, and 1.62% of stance phase duration during ground walking [22]. The control module is a custom electronic board based on a National Instruments System On Module SbRIO-9651, which includes a Real-Time processor and a Field Programmable Gate Array (Xilinx Zynq-7000, 667 MHz), and with additional embedded IMU. The Real-Time processor (100 Hz) implements the algorithms for gait-phase segmentation, the estimation of the biomechanical measurements through the insoles data, and the activation of each vibrotactile unit (VTs). The vGRF and CoP_AP_, as well as the gait event detection and the spatiotemporal parameters of gait, are estimated as described in [17]. The control module is enclosed in a 3D-printed box intended to be worn on the back of the wearer resulting in an overall weight of about 500 g. A graphical user interface runs on a remote computer, connected wirelessly, to initially configure the device and visualize data in real-time.

Lastly, the feedback module comprises 6 VTs positioned equidistantly and symmetrically around the users’ waist in correspondence with the posterior superior iliac spines, lateral iliac crests, and anterior superior iliac spines. Each VT unit can be activated or not according to the purpose of the biofeedback strategy. This placement allows for additional spatial information to be provided exploiting the independent VTs activation (e.g. associating the rearfoot ground contact with the user’s back). The belt is adjustable in size to fit users with different waist circumferences, and the position of the VT units can be easily tuned manually using detachable Velcro strips. Each VT unit contains an eccentric rotating mass motor (Pico Vibe™312-101.005, Precision MicroDrives™) encapsulated in a Polydimethylsiloxane matrix. Aiming to increase the perception of the stimuli and the device’s versatility, each VT can be independently activated and modulated in the vibratory frequency within 100–150 Hz. These frequencies respect both the perceptual capacity of the skin mechanoreceptors responsible for decoding the vibrotactile stimulus and the discriminant capacity of the cerebral cortex relative to the somatosensory system [23].

### Biofeedback strategies

Two biofeedback strategies have been specifically designed to improve the gait patterns of PD subjects. These strategies take into account two typical alterations of the ground reaction force distribution during the stance phase of the gait patterns of PD subjects, namely, the reduced duration of the loading response phase and the reduced duration of the push-off phase in the forefoot. Both alterations are typical of bradykinetic gait and may be present in PD subjects with moderate disability (H&Y ≥ 2) [3].

In the first case, PD subjects typically walk exhibiting a lower peak of vertical ground reaction force during loading response. This alteration causes a distribution of the load on the entire plantar area rather than on the rear foot during the loading response phase [3]. This gait pattern may be due to a reduced ability in maintaining physiological amplitudes in repetitive and automatic movements, or it may be a strategy to compensate for the partial loss of postural control [3]. Based on these considerations, the ‘loading-response enhancement’ strategy entails the activation of the posterior and lateral VTs of each side synchronously with the ipsilateral heel strike until the center of pressure is located on the rearfoot portion. When using this biofeedback strategy, subjects were asked to walk to increase the duration of vibration perceived from the VT units. In order to increase the duration of the vibration, they had to improve the foot-rocker mechanism occurring from heel-strike to mid-stance of the gait cycle. Specifically, subjects approached the ground with the most posterior part of the rearfoot and consequently improving the duration of the loading response and the body weight acceptance in the rearfoot portion [24].

A similar approach was pursued with the ‘push-off enhancement’ strategy. PD subjects show reduced push-off because they exhibit a reduced peak of the ground reaction force in the forefoot portion, a reduced foot-rocker mechanism, and, generally, a lack of hip and knee extension. Consequently, the legs are not adequately accelerated into swing causing shorter stride length and slower gait speed [3]. Hence, the ‘push-off enhancement’ strategy entails the activation of lateral and anterior VTs when the center of pressure of the ipsilateral foot is located on the forefoot portion. Vibrations last until the toe-off event is detected. When using this biofeedback strategy, subjects were requested to walk in a way that increased the duration of the vibration perceived from the VT units. In order to increase the duration of the vibration, they had to increase the foot-rocker mechanism occurring from mid-stance to toe-off, trying to lift the foot only when the center of pressure was at the most anterior portion of the forefoot and consequently increasing the push-off duration and inducing a walking pattern with load distributed on the forefoot [24].

To implement the aforementioned strategies, the BI was programmed to detect in real-time the heel-strike and toe-off events and the location of the center of pressure under the foot sole, in order to identify the load distribution under the rearfoot and forefoot (Fig. 1b). The heel-strike and toe-off events are detected by a pre-set threshold on the real-time vGRF, computed from the pressure-sensitive insole sensor signals as:

$$vGRF= \sum _{i=1}^{16}{F}_{i} {F}_{i}=\left\{\begin{array}{c}f\left(Vi\right), {V}_{i}\le {V}_{noise}\\ 0, {V}_{i}\ge {V}_{noise}\end{array}\right.$$

Where F_i_ are the optoelectronic sensors forces (N), V_i_ are the output voltages (V) and V_noise_ is the noise output voltage threshold (V).

The location of the center of pressure is computed along the anteroposterior direction of the foot sole as:

$${CoP}_{AP}= \frac{{\sum }_{i=1}^{16}({F}_{i}\cdot {w}_{{y}_{i}}\cdot {y}_{i})}{{\sum }_{i=1}^{16}({F}_{i}\cdot {w}_{{y}_{i}})}$$

Where ${y}_{i}$ are the optoelectronic sensors anteroposterior coordinates (cm) and ${w}_{{y}_{i}}$ are the weights (#). When CoP_AP_=0 the vertical load is distributed entirely on the heel, when CoP_AP_=25 cm the load is entirely on the foot tip. Thresholds on the CoP_AP_ were set to identify the rearfoot and forefoot portions (Fig. 1). The vibration intensity of each VT is controlled with a 1 kHz PWM of a 5 V source with a 0-100% duty cycle, as described in detail in [25].

### Participants

Seven subjects were recruited for this case series from the Center for Parkinson’s Disease of Fondazione Don Gnocchi (Milan, Italy) from September 2020 to May 2021. The inclusion criteria were diagnosis of PD, age > 18, Hoehn and Yahr (H&Y) stage between 2 and 4 (the latter criteria was set with the objective to recruit subjects with a mild to moderate bilateral disease and balance impairments that do not compromise the subject’s independence), Mini-Mental State Examination score (MMSE) > 24, ability to walk 10 m independently, stable drug therapy and foot size in between 41 and 43 (EU size). Subjects were excluded if they had deep brain stimulation or any orthopedic, cardiovascular, or respiratory disease. The motor section of the MDS-Unified Parkinson’s Disease Rating Scale (MDS-UPDRS) was used to characterize subjects’ motor symptoms [26]. The New Freezing of Gait Questionnaire (NFOG-Q) has been used to detect subjects affected by freezing of gait.

The study protocol was registered (DGDMF.VI/P/I.5.i.m.2/2019/1297) and approved by the General Directorate of Medical Devices and Pharmaceutical Service of the Italian Ministry of Health and by the ethics committee of the Lombardy region (Milan, Italy). The study was conducted in accordance with the Declaration of Helsinki and all subjects signed an informed consent form.

### Experimental procedures

**Fig. 2:**
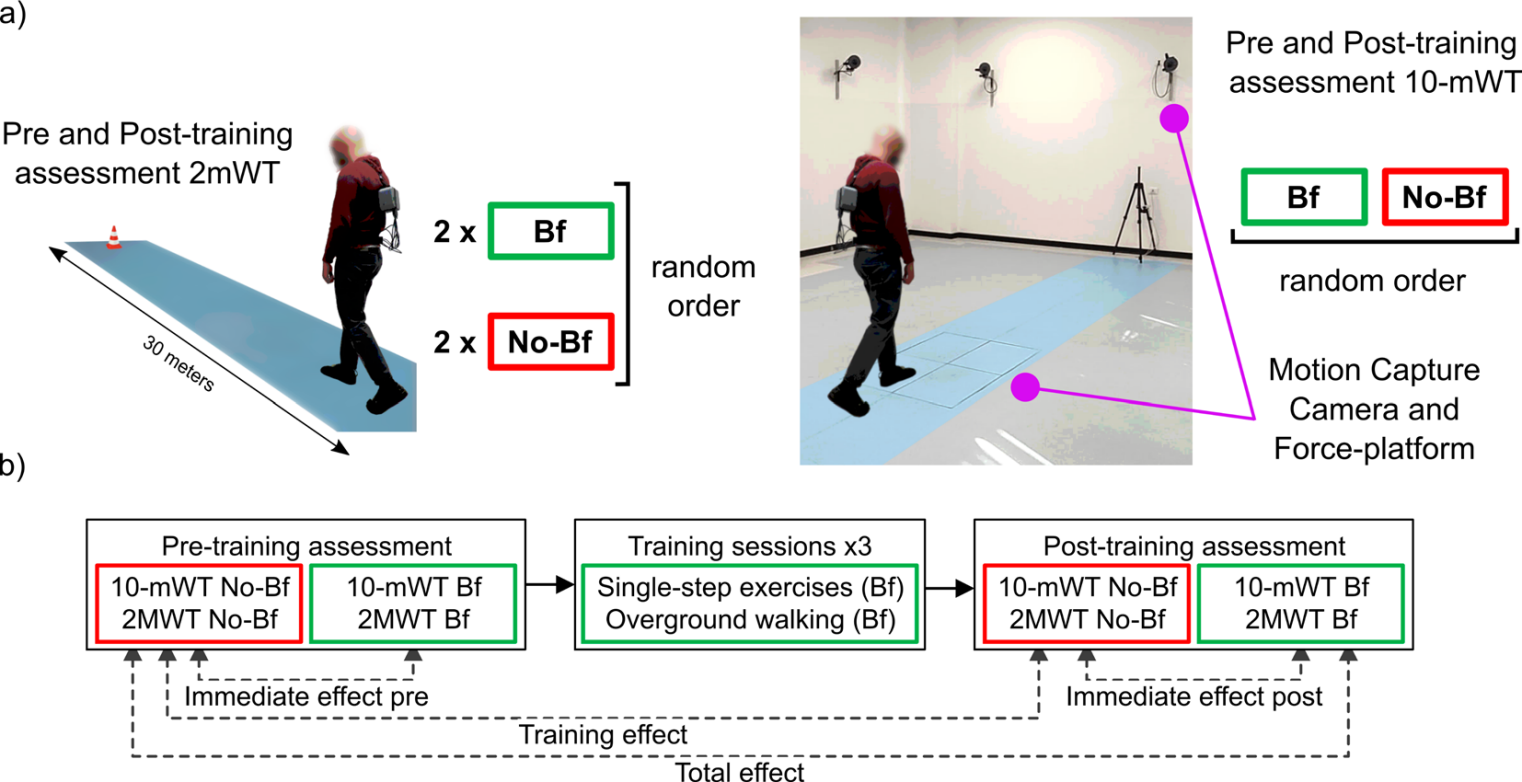
(**a**) Subject performing 2MWT on the left, and during a 10mWT in the gait analysis lab, on the right. (**b**) The experimental procedures and comparative assessments. *Abbreviations* 2MWT, two-minute walk test; 10mWT, 10-meter walkway test; Bf, with biofeedback; No-Bf, without biofeedback

Subjects wore the BI and underwent five experimental sessions on consecutive days, i.e. a pre-training assessment (Pre-trn), three training sessions, and a post-training assessment (Post-trn). Assessments were repeated without (No-Bf) and with (Bf) the activation of the vibrotactile biofeedback. Walking conditions were compared at various time points to evaluate the total effect (TT; Pre-trn_No-Bf versus Post-trn_Bf), the training effect (TE; Pre-trn_No-Bf versus Post-trn_No-Bf), the immediate effect before the training (IE_pre; Pre-trn_No-Bf versus Pre-trn_Bf) and after the training (IE_post; Post-trn_No-Bf versus Post-trn_Bf) (Fig. 2) [27].

Before starting the Pre-trn assessment, each participant wore the BI to verify its appropriateness with body size. In addition, all subjects tried the vibrotactile stimulation to test if their tactile perception was preserved. Finally, each participant performed a short overground walking trial to choose one biofeedback strategy (i.e. ‘loading-response enhancement’ or ‘push-off enhancement’) according to both the physiotherapist assessment and the subject’s preference. In case of a disagreement, the choice was based on the subject’s preference. In all assessments and training sessions, each subject used the same biofeedback strategy that had been previously selected.

### Pre-training and post-training assessment

All subjects were tested in their ON-medication state (one hour after taking their antiparkinsonian medications). The Pre-trn and the Post-trn lasted 30 min and involved two types of gait tests.

First, subjects walked along a 10-meter walkway at their comfortable speed (10-mWT), within a gait analysis laboratory equipped with a motion tracking system and four force platforms (SMART-TD and P6000, BTS S.p.A., Milan, Italy). Force platforms were connected to the motion capture system and placed in the middle of the 10-meter walkway. Optical markers were placed on the subjects according to a simplified LAMB protocol [28]. Data from the motion capture system were processed offline to extract gait speed and step length, together with the vGRF and foot flexion angle profiles. The 10-mWT trials were executed with and without the biofeedback following a random order. Subjects were asked to walk at a comfortable speed and to focus on the biofeedback if activated. They were asked to follow a linear path, looking ahead, without concentrating on hitting the force platform, to avoid conditioning their walking pattern. The 10-mWT was repeated as many times as necessary to record 3 steps from each foot for each experimental condition (No-Bf and Bf). It was decided to record 3 steps from each foot to avoid too much repetition of the 10-mWT and still be able to estimate an average.

Then, subjects performed a two-minute walk test (2MWT) in a 30-meter corridor in a clinical setting and were instructed to walk as fast as they could, safely and without assistance until asked to stop. They were also told that rest breaks were allowed if needed, however, they should start walking again as soon as possible if they felt ready to do so. If they reached the end of the corridor before the two minutes were up, they were told to turn around a cone placed at the end of the corridor and continue walking. The 2MWT ended after two minutes of back and forth walking, and the distance covered was documented [29]. The experiment was conducted four times, two times with biofeedback and two times without, with conditions set in random order. The insoles were used to collect and save plantar pressure data. The strides taken while turning during the execution of 2MWT were identified and labeled, and not included in the analysis. Plantar pressure data from the instrumented insoles were analysed offline to extract temporal parameters (cadence, stride duration, double support, stance, single support, and swing time) and to compute the temporal gait symmetry, also known as the symmetry index (SI), defined as the ratio between the duration of the stance phase computed on both sides [30]. During the assessment, experimenters informed subjects of the presence or absence of the biofeedback without giving any verbal instruction on gait quality to avoid bias. At the end of the Post-trn session, all subjects completed the System Usability Scale (SUS) to assess their subjective perception of the device’s usability [31].

### Biofeedback training sessions

After the Pre-trn assessment, subjects performed three training sessions of 40 min to reinforce their practice with the BI and the biofeedback strategy chosen. Each training session included: (i) an explanation about the rationale and the working principle of the selected biofeedback strategy; (ii) repeated strikes of the heel or forefoot on the ground in sitting position to ensure subjects understood the relation between the plantar pressure and the activation of the VTs; (iii) repeated step in standing position to learn the association between plantar pressure loading and duration of the vibratory stimulus (e.g. during the loading response strategy the duration of the vibratory stimulus increased if they improve the foot rocker mechanism occurring from the heel-strike to the mid-stance); and (iv) overground walking trials asking subjects to focus on the activation of a rhythmical and symmetrical pattern of vibrations perceived at waist level. According to the biofeedback strategy selected, subjects were instructed to modify their walking pattern to increase the duration of the vibrotactile stimulation, therefore improving either the foot-rocker mechanism during the loading response or during the push-off phase.

### Statistics

A descriptive analysis of all variables was carried out. Considering gait parameters, the average value among all steps of a trial was calculated and reported for each subject. In order to objectively measure the change in the participants’ gait parameters, the changes of each subject were considered individually in all the conditions (TT, TE, IE_pre, IE_post) as required by the case series reporting. A qualitative kinetic analysis was performed for each subject on vGRF curves: the magnitude and timing of the loading response and push-off peaks were compared in all conditions taking the vGRF curves of healthy subjects as reference (Fig. 4, Supplementary materials). A qualitative kinematic analysis was performed for each subject on foot flexion angles: The dorsiflexion angles during the heel-strike and the plantarflexion angles from pre-swing to the swing phase of gait were compared in all conditions using the trajectory of healthy subjects as reference (Fig. 4, Supplementary materials). An explorative analysis was computed on the TT, TE, IE_pre, and IE_post considering all subjects. Q-Q plots and Shapiro-Wilk test revealed that data were not normally distributed. Due to the non-normal distribution of the data, medians and interquartile ranges were computed for each condition and the 2-sided Wilcoxon sign-test (*p* < 0.05) was used to verify the effect of the biofeedback device on the whole group. Since we performed a pre-planned comparison and given the explorative nature, no post-hoc analysis was carried out. Finally, we calculated the SUS score adding up the converted responses and multiplying that total by 2.5 for each subject. The SUS final score was interpreted according to the literature [31]. The number of adverse events were recorded and categorized when occurred. All analyses were performed using SPSS software, version 26 (IBM, NY, USA), and Microsoft Excel 2019.

## Results

### Characteristics of the sample

Seven subjects completed the protocol (Table 1). All subjects scored 2.5 or 3 points on the H&Y scale indicating mild to moderate disease with postural instability [32]. According to the MDS-UPDRS motor section, five subjects presented mild to moderate motor symptoms (< 32 points) while two subjects showed moderate to severe motor symptoms (> 58 points) [33]. The NFOG-Q revealed that five out of seven subjects showed FOG. Subjects #1,2,3,4,5 used the ‘*loading-response enhancement*’ (LR) strategy, while subjects #6 and #7 used the ‘*push-off enhancement*’ (PO) strategy.

**Table 1:** Demographic characteristics of the subjects, mean values, and standard deviations

Subjects	Age(years)	Gender	Disease duration(years)	H&Y	MDS-UPDRS	NFOG-Q	MMSE	Shoe Size(EU)	Biofeedback strategy
Subject 1	59	Male	9	3	33	7	30	41	LR
Subject 2	72	Male	4	3	60	8	26	43	LR
Subject 3	76	Male	6	2.5	35	0	27	43	LR
Subject 4	73	Male	26	3	53	20	28	42	LR
Subject 5	59	Male	2	2.5	56	16	28	43	LR
Subject 6	75	Male	4	2.5	39	0	29	42	PO
Subject 7	79	Male	11	3	68	19	29	41.5	PO
Mean ± SD	70.4 ± 8.1		12 ± 5.6	2.7 ± 0.3	49.1 ± 13.5	10 ± 8.4	28.1 *± 1.4*	42.2 *± 0.8*	

*Abbreviations* H&Y, Hoehn and Yahr; MDS-UPDRS, Movement Disorder Society-Unified Parkinson’s Disease Rating Scale; NFOG-Q, New Freezing of Gait Questionnaire; MMSE, Mini-mental state examination; EU, European; SD, Standard deviation; LR, Loading response; PO, Push-off

### Pre-training and post-training assessment results

Results of the gait tests performed in the Pre-trn and Post-trn assessment sessions are presented below. Gait parameters measured during each test (10mWT or 2MWT) are presented separately considering the walking conditions and the time points explained in the experimental procedures (Fig. 2). For each test first the TT and TE results are presented, then the IE results for both Pre-trn and Post-trn sessions.

### Gait parameters during 10mWT

Results of the 10mWT are reported in Table 2 analysing in detail all the conditions (TT, TE, IE_pre, IE_post). vGRF curves and Foot flexion angles data are reported in (Supplementary materials Fig. 4).

**Table 2:** Outcomes from 10-mWT

Outcome measures			Pre-trn		Post-trn		Statistical analysis wilcoxon sum rank test (*p*-value)
		Biofeedback strategy	No-Bf(1)	Bf(2)	No-Bf(3)	Bf(4)	
Gait speed (m/sec)	*Subject 1* *Subject 2* *Subject 3* *Subject 4* *Subject 5* *Subject 6* *Subject 7* ***Median(1°,3°)***	LR LR LR LR LR PO PO	0.46 0.47 0.78 0.72 1,06 0,72 0,74 **0.72(0.59,0.72)**	0.32 0.42 0.68 0.55 1,03 0,53 1,07 **0.55(0.48,0.85)**	0.75 0.72 0.96 1.06 1.35 1.02 0.62 **0.96(0.73,1.04)**	0.50 0.79 0.95 0.99 1.24 0.98 0.58 **0.95(0.69,0.98)**	IE_pre (0.237)
							IE_post (0.128)
							TE (0.028)*
							TT (0.043)*
Step length (m)	*Subject 1* *Subject 2* *Subject 3* *Subject 4* *Subject 5* *Subject 6* *Subject 7* ***Median(1°,3°)***	LR LR LR LR LR PO PO	0.87 0.72 0.94 0.81 1.16 0.81 0.96 **0.87(0.81,0.96)**	0.77 0.87 0.98 0.77 1.18 0.91 1.17 **0.91(0.82,1.07)**	1.1 0.85 1.07 1.08 1.37 1.02 0.87 **1.1(0.95,1.09)**	0.94 0.98 1.19 1.09 1.33 1.05 0.91 **1.05(0.96,1.14)**	IE_pre (0.31)
							IE_post (0.612)
							TE (0.028)*
							TT (0.028)*

*Abbreviations* 10-mWT, 10-meter walkway test; Pre-trn, pre-training assessment; Post-trn, post-training assessment; LR, Loading response; PO, Push-off; No-Bf, without biofeedback; Bf, with biofeedback. IE_pre (Immediate effect Pre, 1vs2), IE_post (Immediate effect Post, 3vs4), TE (Training Effect, 1vs3), TT (Total Effect, 1vs4), * *p* < 0.05

#### Total and training effects

Six subjects (#1,2,3,4,5 using the LR strategy, and #6 using the PO strategy) increased gait speed and step length as TT and TE, while subject #7 (using the PO strategy) showed a reduction in both gait parameters as TT and TE. The exploratory analysis on the whole group revealed a significant improvement in gait speed as TT (Pre-trn_No-Bf: 0.72(0.59,0.72) m/sec; Post-trn_Bf: 0.95(0.69,0.98) m/sec; *p* = 0.043) and TE (Pre-trn_No-Bf: 0.72(0.59,0.72) m/sec; Post-trn_No-Bf: 0.96(0.73,1.04) m/sec; *p* = 0.028). Similar improvements were detected in step length as TT (Pre-trn_No-Bf: 0.87(0.81,0.96) meters; Post-trn_Bf: 1.05(0.96,1.14) meters; *p* = 0.028) and TE (Pre-trn_No-Bf: 0.87(0.81,0.96) meters; Post-trn_No-Bf: 1.1(0.95,1.14) meters; *p* = 0.028).

The vGRF profiles showed qualitative improvements in five subjects (#2,3,4,5,6) due to TT, and six subjects (#1,2,3,4,5,6) due to TE. For both TT and TE, foot flexion angles showed improved dorsiflexion and plantarflexion in six subjects (#1,2,3,4,5,6). Subject #7 showed no improvement in vGRF profiles and foot flexion angles as TT and TE.

#### Immediate effect

At Pre-trn, six subjects (#1,2,3,4,5,6) decreased their gait speed when the biofeedback device was activated while subject #7 showed an increase in gait speed of 0.33 m/sec. Five subjects (#2,3,5,6,7) increased step length as IE while subjects #1 and #4 decreased step length of 0.1 m and 0.04 m respectively. Improved vGRF was detected in subject #7 and improved feet’s kinematics were detected in three subjects (#2,6,7) as IE of the device.

At Post-trn, six subjects (#1,3,4,5,6,7) decreased their gait speed as IE while subject #2 showed improvement in gait speed of 0.07 m/sec. Five subjects (#2,3,4,6,7) increased step length as IE, while subjects #1 and #5 decreased step length of 0.16 and 0.11 m, respectively. An improved vGRF curve was observed in subject #2 and improved feet’s kinematics was observed in subjects #2 and #3 as IE of the device.

### Gait parameters during 2MWT

**Fig. 3:**
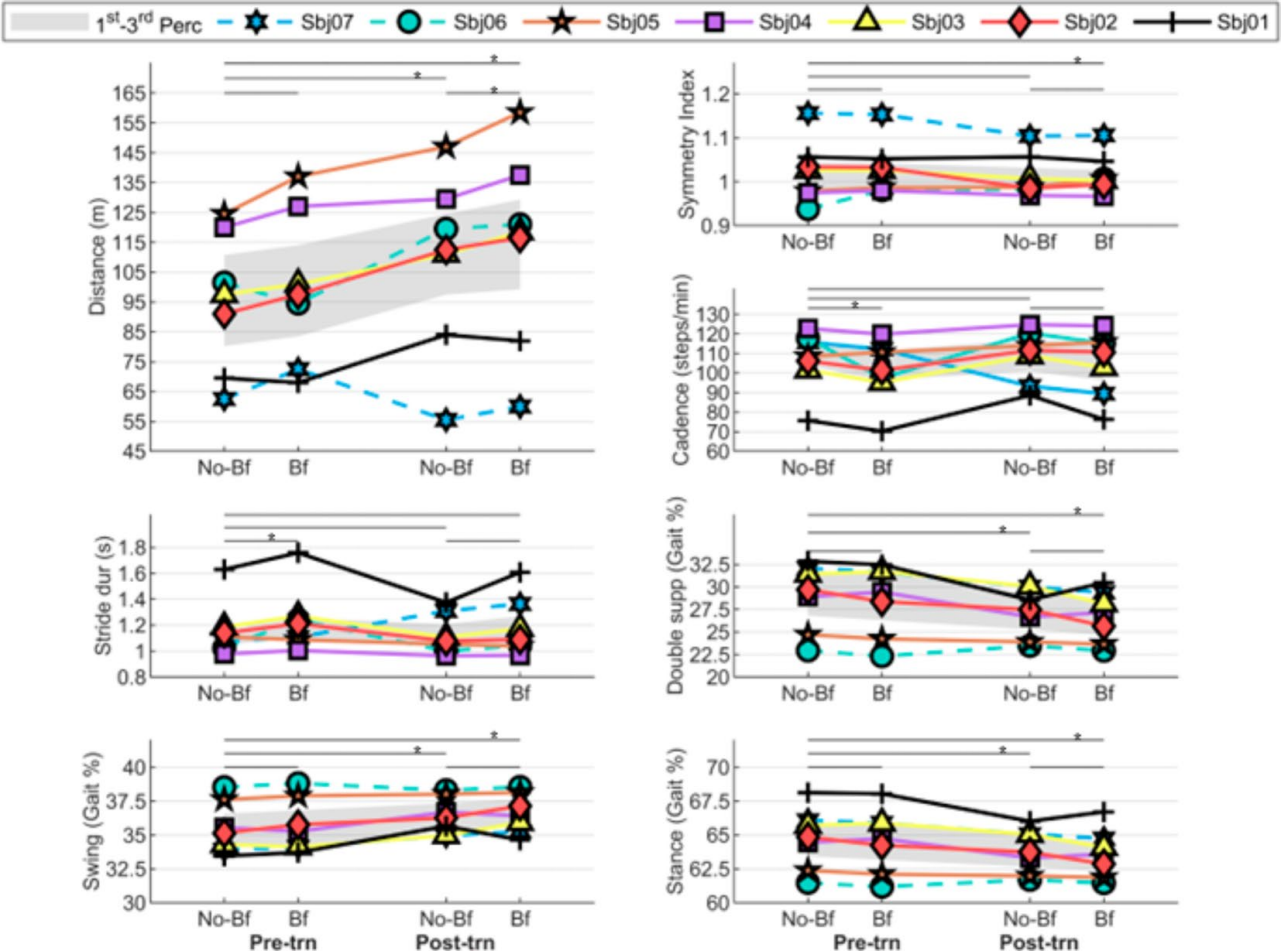
Line charts of gait parameters for each subject during the 2MWT. *Abbreviations* Pre-trn, pre-training assessment; Post-trn, post-training assessment; No-Bf, without biofeedback; Bf, with biofeedback; Sbj, Subject. * *p* < 0.05

Results of the 2MWT are reported in Fig. 3. All the conditions (TT, TE, IE_pre, IE_post) are also reported in (Supplementary materials Table 3).

#### Total and training effects

Six subjects (#1,2,3,4,5 using the LR strategy, and #6 using the PO strategy) increased walking distance as TT and TE while five subjects (#1,2,3,4,5) slightly increased cadence as TT. Subject #6 showed a reduction in cadence of 2.6 steps/min as TT despite an increase of 19.5 m in walking distance. Subject #7 (using the PO strategy) showed a reduction in cadence of 26.3 steps/min as TT despite a decrease of 2.5 m in walking distance. Improvements in walking distance showed a significant TT (Pre-trn_No-Bf: 97.5 (80.3,110.8) meters; Post-trn_Bf: 118.5(99.3,129.3) meters; *p* = 0.028) and TE (Pre-trn_No-Bf: 97.5 (80.3,110.8) meters; Post-trn_No-Bf: 112.5(97.5,124.5) meters; *p* = 0.028) considering the median value of the whole group.

Considering the gait cycle sub-phases, six subjects (#1,2,3,4,5,7) decreased the double support phase and the stance phases duration as TT and TE, while increasing the single support phase and the swing phase duration. The same parameters remained unchanged for subject #6 as TT while as TE subject #6 showed an increase of 0.2% and 0.6% in the stance phase and double support phase duration respectively, and a reduction of 0.2% in the swing phase duration. The exploratory analysis on the whole group revealed a significant decrease of the double support duration as TT (Pre-trn_No-Bf: 29.7(26.8,31.7) %; Post-trn_Bf: 27.2(24.6,28.7) %; *p* = 0.018) and TE (Pre-trn_No-Bf: 29.7(26.8,31.7) %; Post-trn_No-Bf: 27.5(25.3,29.3) %; *p* = 0.028). Significant improvements have also been detected in the other gait cycle sub-phases as reported in (Supplementary materials Table 3).

All subjects showed an asymmetrical walking pattern at Pre-trn and five subjects (#2,3,5,6,7) improved their gait symmetry after the training as TT and TE. The SI remained unchanged for subject #4 both as TT and TE while subject #1 showed a slight worsening of 0.1 point in the SI as TE. Improvement of the SI showed a significant TT considering the median value of the whole group (Pre-trn_No-Bf: 1.03(0.98,1.04); Post-trn_Bf: 1(0.99,1.03); *p* = 0.028).

#### Immediate effect

At Pre-trn, five subjects (#2,3,4,5,7) increased walking distance as IE when the biofeedback device was activated while in subjects #1 and #6 walking distance was reduced by 1,5 m and 7 m respectively. Six subjects (#1,2,3,4,6,7) reduced cadence while in subject #5 the cadence was increased by 2.2 steps/min. The analysis on the whole group revealed a significant decrease in cadence as IE of the device (Pre-trn_No-Bf: 108(103.8,116.7) step/min; Pre-trn_Bf: 101.4(96.3,111.4) step/min; *p* = 0.028).

At Pre-trn five subjects (#1,2,5,6,7) decreased the double support phase and the stance phase duration while increasing the swing phase duration as IE. Conversely, two subjects (#3,4) increased the double support phase, the stance phase, and decreased the swing phase duration. Finally, three subjects (#4,5,6) improved their gait symmetry while subject #1 showed a 0.1 worsening of the SI and in three subjects (#2,3,7) the SI remained unchanged.

At Post-trn, six subjects (#2,3,4,5,6,7) increased walking distance while subject #1 walked 2 m less when the biofeedback device was activated. The exploratory analysis revealed a significant improvement in walking distance as IE (Post-trn_No-Bf: 112.5(97.5,124.5) meters; Post-trn_Bf: 118.5(99.3,129.3) meters; *p* = 0.043). Six subjects (#1,2,3,4,6,7) decreased cadence while in subject #5 cadence was increased by 1.8 step/min. Five subjects (#2,3,5,6,7) decreased the double support phase, stance phase and increased the swing phase duration as IE of the device. Conversely, subjects #1 and #4 increased the double support phase and stance phase while reducing the swing phase duration. Four subjects (#1,2,3,6) improved gait symmetry while subject #7 showed a 0.1 worsening of the SI and in subjects #4 and #5 the gait symmetry remained unchanged.

#### Usability and safety

All subjects completed the protocol with no adverse events or drop-outs. The SUS scores measured at the end of the Post-trn assessment were analyzed considering 68 as a threshold score for acceptability and 80.3 as the threshold for a full positive evaluation [31]. For five subjects the SUS score fell between 60 and 80.3 points, while for two subjects SUS resulted higher than 80.3. For each participant, the SUS scores are shown in Table 3.

**Table 3:** System usability scale (SUS). The SUS scores range from 1 (“strongly disagree”) to 5 (“strongly agree”)

Questionnaire Item	Subject 1	Subject 2	Subject 3	Subject 4	Subject 5	Subject 6	Subject 7
I think that I would like to use this system frequently	5	5	5	4	3	5	4
I found the system unnecessarily complex.	4	1	1	2	2	2	2
I thought the system was easy to use.	5	5	4	4	5	5	4
I think that I would need the support of a technician to use the system.	3	4	5	1	4	5	4
I found the various functions in this system were well integrated.	5	5	5	4	4	5	2
I thought there was too much inconsistency in this system.	1	1	1	1	2	2	1
I imagine that most people would learn to use this system very quickly.	4	5	5	5	5	5	4
I found the system very cumbersome to use.	1	1	1	1	1	2	2
I felt very confident using the system.	5	5	4	5	4	5	4
I needed to learn a lot of things before I could get going with this system.	4	3	4	1	1	5	1
**SUS score**	**77.5**	**87.5**	**77.5**	**82.5**	**77.5**	**77.5**	**77.5**

## Discussion

### The system architecture of the BI

The system architecture of the BI is similar to other wearable vibrotactile biofeedback devices shown in previous studies [9, 14]. These devices allow to measure both spatial and temporal parameters of gait and both kinetic and kinematic information. However, even if the current prototype presents an embedded IMU in the control module placed at the trunk this has not yet been implemented to improve the functions of the device. Indeed, one limitation of this study resides in the missing information about trunk movements in the medial-lateral plane, which is crucial for postural stability during gait. Further improvements in the wearability of the BI might be made to use the IMU data for stability analysis, even though the BI control module offers greater portability and facility of use in different environments compared to other devices [14]. Regarding the feedback module, eccentric rotating mass motors are a common choice for vibrotactile devices due to their low power consumption and small size. However, the number of vibrotactile units is a determining factor for human perception of the stimuli, and by reducing the number of units, the users’ perception of the vibratory stimulus looks to increase [34]. These BI characteristics are relevant to re-integrating the controlled gait pattern into the subjects’ motor system, associating the stimulus with pre-defined gait phases. A possible limitation of this approach is that the somatosensory cortex is saturated when it reaches a plateau of relatively low frequencies, reducing the perception of a semi-continuous stimulus over time [14]. Nevertheless, the vibratory stimulus and the position of the actuators on the waist in people with PD are supported in the literature [35]. Finally, an important feature of the BI is the possibility to customize the vibrations, allowing modification of the activation strategies if the subject adapts to the stimulus.

### Change in gait parameters, usability, and safety

The results of this case series showed a positive evaluation of the device’s usability and safety in clinical scenarios. However, subjects’ usability evaluations should be interpreted considering the supervised use of the BI by trained personnel.

Clinically, this device could be used as both an assessment and a biofeedback tool. Considering the assessment, the BI was used to extract gait parameters when performing a clinical walking test such as the 2MWT. This is a widely used test in clinical practice that usually provides a measurement of the distance walked in two minutes along a 30 m corridor. Due to the space required to perform the test, this cannot be easily performed in gait analysis laboratories. The use of the BI during the execution of this test allows to obtain relevant gait parameters about which clinicians would normally have no information.

Regarding the effect of the BI on the gait pattern, improvements were registered as IE_pre, IE_post, TE, and TT in gait parameters relevant to PD, such as the walking distance, stride length, cadence, gait speed, and double-support phase duration. However, the immediate effect of the device was subtle, as in some subjects the gait speed and step length decreased when the biofeedback was presented as an immediate effect, in particular during the Pre-trn phase. These results are likely due to a motor learning process, since subjects were new to the device, and a cognitive effort was required to understand the task request. Even if all subjects did not show cognitive impairments (MMSE > 24), it is possible that attentional resources and cognitive strategies were affected, in agreement with the findings of a fronto-striatal deficit in this population [36]. Moreover, considering its exploratory nature, the whole group analysis revealed that the immediate effect was statistically significant in a small minority of the 9 outcomes reported. In particular, IE_pre was only significant for cadence and stride duration and IE_post only for the distance walked.

The most relevant improvements were recorded as TE and TT, as a period of biofeedback training appears to be necessary to achieve clinically significant results. Indeed, the three-day training program presented in this article led to gait speed improvement and a 10% increment in the distance walked in the 2MWT, corresponding to the minimal clinically important difference in the neurological population [37]. On the other hand, gait speed improvement may not necessarily be a positive outcome in people with PD since slower gait speed may be partially compensatory due to reduced balance. As a slower gait combined with shorter strides is a clinical predictor of future falls [38], changes in gait speed alone could be dangerous if not accompanied by improvements in other important aspects of gait, such as stride length. However, our results showed that gait speed improvements occurred together with stride length, vGRF profiles, and foot flexion angle improvements, suggesting a positive change in multiple gait features. Hence, gait speed improvement may be interpreted as a positive outcome, considering also gait speed as a valid measure to predict community ambulation in people with PD [39].

These results are in line with previous literature showing positive effects in spatiotemporal gait parameters of step-synchronized biofeedback in PD [15, 16]. In addition, this study found preliminary, qualitative improvements in kinetic and kinematic profiles that have not been highlighted in previous studies. The reappearance of more physiological kinetic and kinematic profiles, along with improvements in functional tests, may suggest a motor recovery in most subjects [40].

Even if five out of seven subjects reported FOG at the pre-training assessment, it was not possible to establish the impact of the vibrotactile biofeedback on this symptom because no relevant FOG episodes were measured during any of the experimental procedures. FOG absence could be explained by the inadequacy of the experimental set-up (not suitable for stimulating FOG), moreover, all subjects were tested during their ON-medication state. One of the factors that could have caused a freezing episode was the turning required during the 2MWT at the end of the 30-meter corridor, but no episodes occurred here either.

Future studies should verify the impact of the BI on FOG using an experimental set-up that includes obstacles, turning and narrow spaces, and with different subjects’ condition (e.g. during the levodopa wearing-off phase). Considering the biofeedback strategies, both seemed effective in improving the gait pattern. Both strategies were aimed at improving the foot-rocker mechanism either by improving toe-off during the push-off phase or by improving heel strike during the initial load response phase. No relevant differences were observed between the five subjects who used the ‘loading-response enhancement’ strategy and the two subjects who used the ‘push-off enhancement’ strategy. Indeed, subject #6, who used the ‘push-off enhancement’, presents similar results to the other five subjects in the main outcomes (such as distance walked and gait speed). This might suggest that the differences between subject #7 and the rest of the group are due to different causes rather than the type of strategy used. Furthermore, it should be emphasized that the choice of strategy to be used was mainly based on the subject’s preference. Novel strategies could be designed in future studies to provide even more personalized training programs.

## Limitations of the study

This case series belongs to the “proof-of-concept” studies that are usually small and preliminary to justify further complex studies [41]. From a methodological point of view, the proposed experimental procedures align with previous work [14] comparing subjects’ motor behavior in session with and without the vibrotactile stimulus. In addition, we provided a short training phase to reinforce subjects’ practice with the BI and to have a more accurate usability evaluation. However, despite its proof-of-concept nature, this study presents several limitations that must be highlighted. Major limitations are the recruitment of a small sample of male subjects and the absence of a follow-up assessment. These limitations compromise our ability to generalize the results to a larger population and to extrapolate conclusions on the long-term effects and retention of biofeedback training. Moreover, the lack of a control group and blinded assessment does not allow for clear attributing of the observed improvement in gait features to the biofeedback-based intervention. Indeed, spontaneous motor learning and context-specific adaptation and/or habituation cannot be ruled out as a potential mechanism to explain the findings. Nevertheless, it is worth noting that the study subjects obtained clinically significant results despite only three days of biofeedback training, while the training duration in randomized clinical trials involving wearable devices in the neurological population usually ranged from 10 to 30 sessions over 2 months [9]. Future randomized controlled trials should be designed to test the feasibility and efficacy of a home-based rehabilitation program using the BI compared to conventional physiotherapy, including both male and female subjects and a minimum of 3 months follow-up.

### Future perspectives

Considering the device’s characteristics, given its prototype nature, future developments will focus on reducing the dimension of the control module and increasing the portability. Nevertheless, the device was designed to open the possibility of home-based rehabilitation. Consequently, the device is required to be set up for the user only during the first session, with the help of the physiotherapist in the rehabilitation scenario, no other settings are required to use the biofeedback strategies in everyday use. With this concept, the physiotherapist is able to download the data from the device remotely and evaluate the frequency of use of the device and the progress of rehabilitation.

Results from this study suggest that people with PD could easily learn how to use the BI after a short training period. After appropriate training, it could be used at home or outdoors to assist gait providing simultaneously real-time data that will subsequently be displayed to the clinicians to monitor progress. Monitoring could be repeated over time according to clinical needs (several times a day, once a week, a month, etc.).

## Conclusion

In conclusion, this case series suggests the usability and safety of a wearable biofeedback device measuring gait parameters in people with PD as part of a clinical walking test and delivering step-synchronized vibrotactile biofeedback. PD subjects showed short-term improvements in functional tests and instrumental assessment suggesting that the biofeedback device could stimulate motor recovery. Future trials should investigate the possibility of using this wearable biofeedback device as an instrument for clinical assessment and rehabilitation in clinical or remote environments.

### Electronic supplementary material

Below is the link to the electronic supplementary material.

Supplementary Material 1

Supplementary Material 2

## Data Availability

The data supporting the conclusions of this article are included within the article and its additional files.

## Abbreviations

2MWT: two-minute walk test
10mWT: 10-meter walkway test
BI: Wearable Vibrotactile Bidirectional Interface
Bf: with biofeedback
No-Bf: without biofeedback
CoP: Center of pressure
EU: European; FOG, Freezing of Gait
FPGA: Field Programmable Gate Array
H&Y: Hoehn and Yahr
IE_post: Immediate effect Post
IE_pre: Immediate effect Pre
IMU: Inertial Measurement Units
MAE: Median Absolute Error
MDS-UPDRS: Movement Disorder Society-Unified Parkinson’s Disease Rating Scale
MMSE: Mini-mental state examination
NFOG-Q: New Freezing of Gait Questionnaire
PD: Parkinson’s Disease
Post-trn: post-training assessment
Pre-trn: pre-training assessment
SD: Standard deviation
SI: Symmetry Index
SUS: System Usability Scale
TE: Training Effect
TT: Total Effect
vGRF: vertical ground reaction force
VT: Vibrotactile Unit
