# Supplementary material for: Wearable biofeedback device to assess gait features and improve gait pattern in people with parkinson’s disease: a case series
Source: J Neuroeng Rehabil. 2024 Jun 26;21:110. doi: 10.1186/s12984-024-01403-z (PMC11202340; doi:10.1186/s12984-024-01403-z)
Supplement: Supplementary file 1 — Supplementary Material 1 [file 12984_2024_1403_MOESM1_ESM.pdf]

Table 3. Supplementary materials: **Outcomes 2-MWT. Abbreviations: 2MWT, two-minute walk test; Pre-trn, pre-training assessment; Post-trn, post-training assessment; LR, Loading response; PO, Push-off; No-Bf, without biofeedback; Bf, with biofeedback. IE_pre (Immediate effect Pre, 1vs2), IE_post (Immediate effect Post, 3vs4), TE (Training Effect, 1vs3), TT (Total Effect, 1vs4), * p<0.05.**

Outcome measures		Biofeedback strategy	Pre-trn		Post-trn		Statistical Analysis Wilcoxon sum rank Test (*p*-value)
			No-Bf(1)	Bf(2)	No-Bf(3)	Bf(4)	
**Distance (m)**	*Subject 1*	LR	69.5	68.0	84.0	82.0	IE_pre (0.150)
	Subject 2	LR	91.0	97.5	112.5	116.5	
	Subject 3	LR	97.5	101.0	111.0	118.5	IE_post (0.043)*
	Subject 4	LR	120.0	127.0	129.5	137.5	
	Subject 5	LR	124.5	137.0	147.0	158.5	TE (0.028)*
	Subject 6	PO	101.5	94.5	119.5	121.0	
	Subject 7	PO	62.5	72.5	55.5	60.0	TT (0.028)*
	Median(1°,3°)		**97.5(80.3,110.8)**	**97.5(83.3,114)**	**112.5(97.5,124.5)**	**118.5(99.3,129.3)**	
**Cadence (step/min)**	*Subject 1*	LR	75.7	70.3	88.5	76.3	IE_pre (0.028)*
	Subject 2	LR	106.2	101.4	111.5	110.7	
	Subject 3	LR	101.5	95.2	108.8	102.8	IE_post (0.063)
	Subject 4	LR	122.7	119.8	124.7	124.1	
	Subject 5	LR	108.4	110.6	113.9	115.7	TE (0.23)
	Subject 6	PO	117.8	97.4	120.3	115.2	
	Subject 7	PO	115.7	112.1	93.2	89.4	TT (0.61)
	Median(1°,3°)		**108(103.8,116.7)**	**101.4(96.3,111.4)**	**111.5(101,117.1)**	**110.7(96.1,115.5)**	
**Stride duration (seconds)**	*Subject 1*	LR	1.63	1.75	1.37	1.60	IE_pre (0.028)*
	Subject 2	LR	1.14	1.21	1.08	1.09	
	Subject 3	LR	1.19	1.27	1.11	1.17	IE_post (0.063)
	Subject 4	LR	0.98	1.00	0.96	0.97	
	Subject 5	LR	1.11	1.09	1.05	1.04	TE (0.176)
	Subject 6	PO	1.02	1.24	1.00	1.04	
	Subject 7	PO	1.08	1.11	1.31	1.36	TT (0.499)
	Median(1°,3°)		**1.11(1.05,1.16)**	**1.21(1.09,1.25)**	**1.08(1.02,1.2)**	**1.09(1.04,1.26)**	
**Stance (gait phase%)**	*Subject 1*	LR	68.1	68.0	66.0	66.7	IE_pre (0.237)
	Subject 2	LR	64.8	64.2	63.7	62.8	
	Subject 3	LR	65.7	65.9	65.0	64.1	IE_post (0.31)
	Subject 4	LR	64.4	64.7	63.3	63.6	
	Subject 5	LR	62.4	62.1	62.0	61.9	TE (0.028)*
	Subject 6	PO	61.5	61.2	61.7	61.5	
	Subject 7	PO	66.1	65.9	65.1	64.7	TT (0.018)*
	Median(1°,3°)		**64.8(63.4,65.9)**	**64.3(63.2,65.9)**	**63.7(62.6,65.1)**	**63.5(62.3,64.4)**	
**Double support (gait phase%)**	*Subject 1*	LR	32.9	32.5	28.6	30.5	IE_pre (0.176)
	Subject 2	LR	29.7	28.4	27.5	25.7	
	Subject 3	LR	31.4	31.7	30.1	28.1	IE_post (0.31)
	Subject 4	LR	28.9	29.4	26.7	27.2	
	Subject 5	LR	24.7	24.2	23.9	23.6	TE (0.028)*
	Subject 6	PO	22.9	22.3	23.5	22.9	
	Subject 7	PO	32.1	31.7	30.0	29.4	TT (0.018)*
	Median(1°,3°)		**29.7(26.8,31.7)**	**29.4(26.3,31.7)**	**27.5(25.3,29.3)**	**27.2(24.6,28.7)**	
**Swing (gait phase%)**	*Subject 1*	LR	33.4	33.7	35.6	34.6	IE_pre (0.499)
	Subject 2	LR	35.1	35.7	36.3	37.1	
	Subject 3	LR	34.3	34.1	35.0	35.9	IE_post (0.176)
	Subject 4	LR	35.5	35.3	36.7	36.4	
	Subject 5	LR	37.6	37.9	38.0	38.1	TE (0.028)*
	Subject 6	PO	38.5	38.8	38.3	38.5	
	Subject 7	PO	33.9	34.1	34.9	35.3	TT (0.018)*
	Median(1°,3°)		**35.2(34.1,36.6)**	**35.3(34.1,36.8)**	**36.3(35.3,37.4)**	**36.4(35.6,37.6)**	
**Symmetry Index**	*Subject 1*	LR	1.05	1.06	1.06	1.05	IE_pre (0.31)
	Subject 2	LR	1.03	1.03	0.99	1.00	
	Subject 3	LR	1.03	1.03	1.01	1.00	IE_post (0.237)
	Subject 4	LR	0.97	0.98	0.97	0.97	
	Subject 5	LR	0.98	0.99	1.00	1.00	TE (0.063)
	Subject 6	PO	0.94	0.98	0.98	1.01	
	Subject 7	PO	1.15	1.15	1.10	1.11	TT (0.028)*
	Median(1°,3°)		**1.03(0.98,1.04)**	**1.03(0.98,1.04)**	**1(0.99,1.03)**	**1(0.99,1.03)**	
