# Supplementary material for: Wearable biofeedback device to assess gait features and improve gait pattern in people with parkinson’s disease: a case series
Source: J Neuroeng Rehabil. 2024 Jun 26;21:110. doi: 10.1186/s12984-024-01403-z (PMC11202340; doi:10.1186/s12984-024-01403-z)
Supplement: Supplementary file 2 — Supplementary Material 2 [file 12984_2024_1403_MOESM2_ESM.pdf]

Figure 4. Supplementary materials: **vGRF (%Body Weight), Foot flexion angle (degree), Stride Velocity (m/s) and Stride Length (m) at Pre-training (Pre-trn) and Post-training (Post-trn) during 10-mWT.**

Subject 1

Left side | Right side

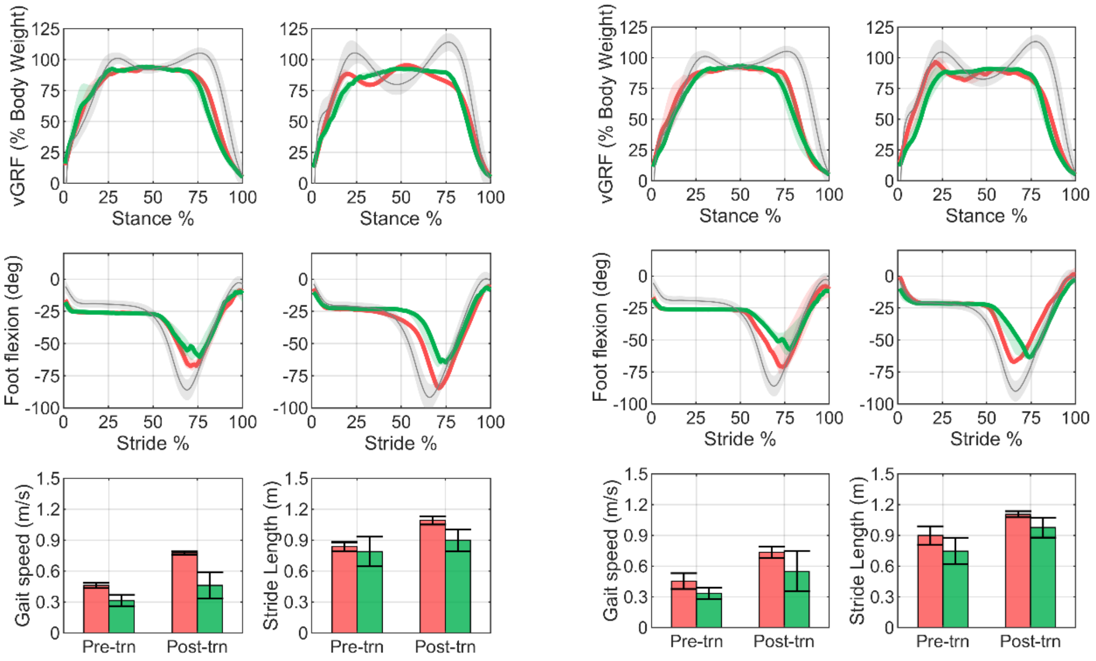

■ Healthy subjects normality band ■ Assessment without Biofeedback ■ Assessment with Biofeedback

Subject 2

Left side | Right side

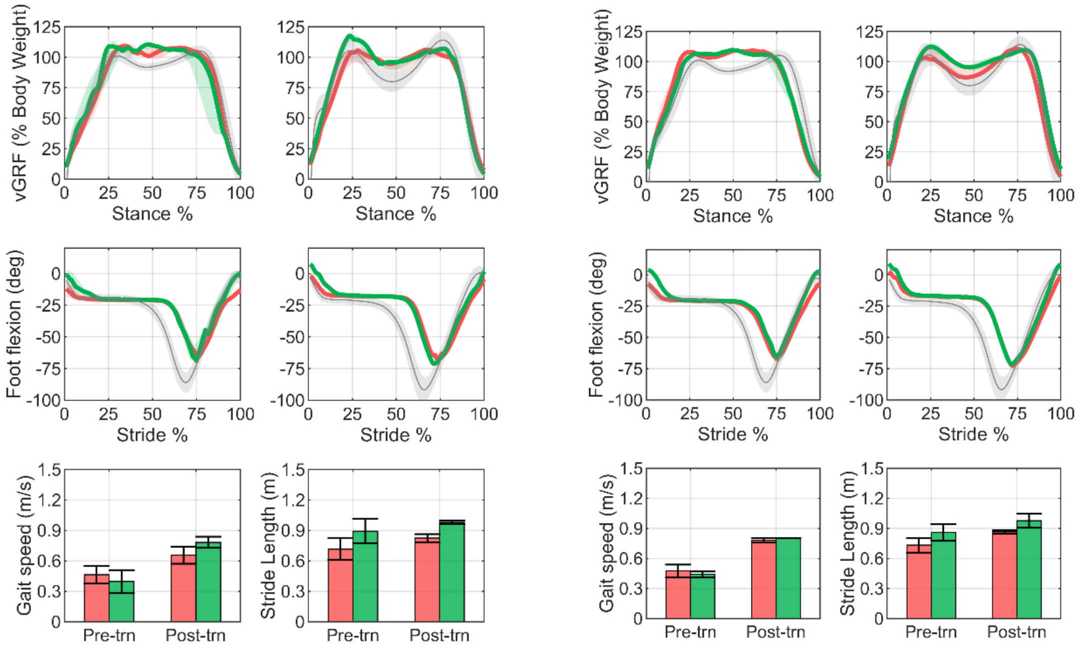

■ Healthy subjects normality band ■ Assessment without Biofeedback ■ Assessment with Biofeedback

Subject 3

Left side

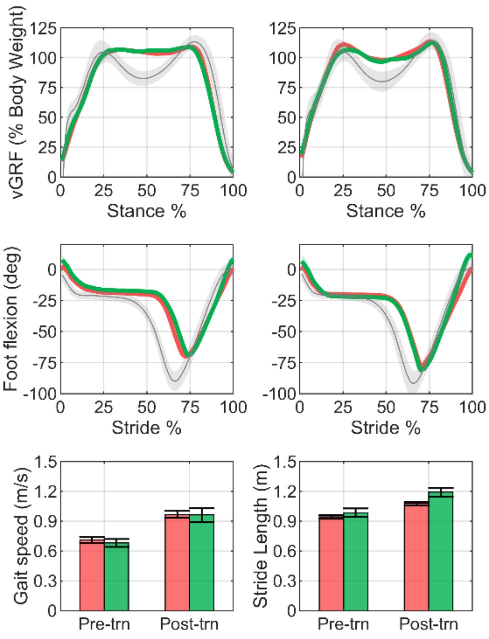

Right side

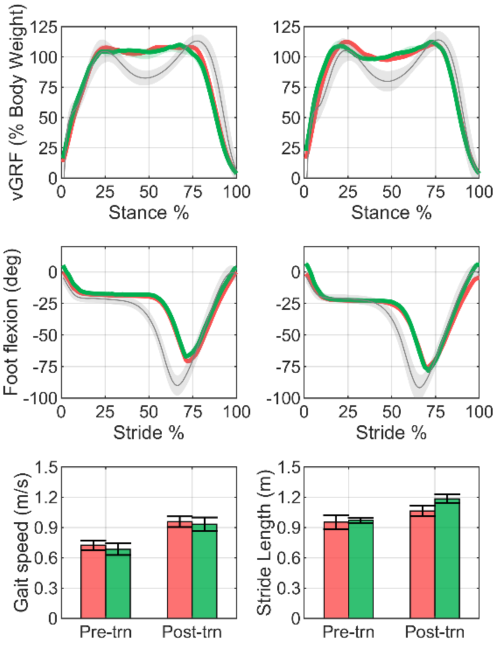

■ Healthy subjects normality band ■ Assessment without Biofeedback ■ Assessment with Biofeedback

Subject 4

Left side

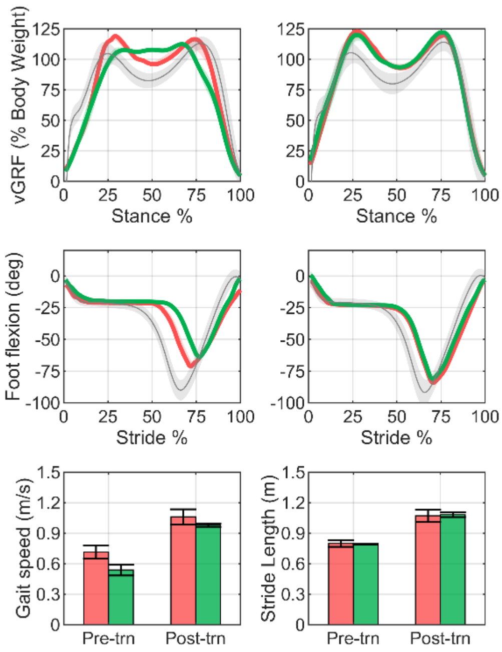

Right side

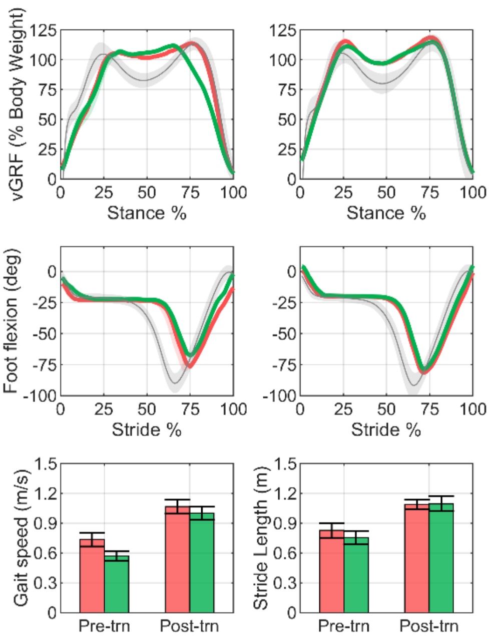

■ Healthy subjects normality band ■ Assessment without Biofeedback ■ Assessment with Biofeedback

Subject 5

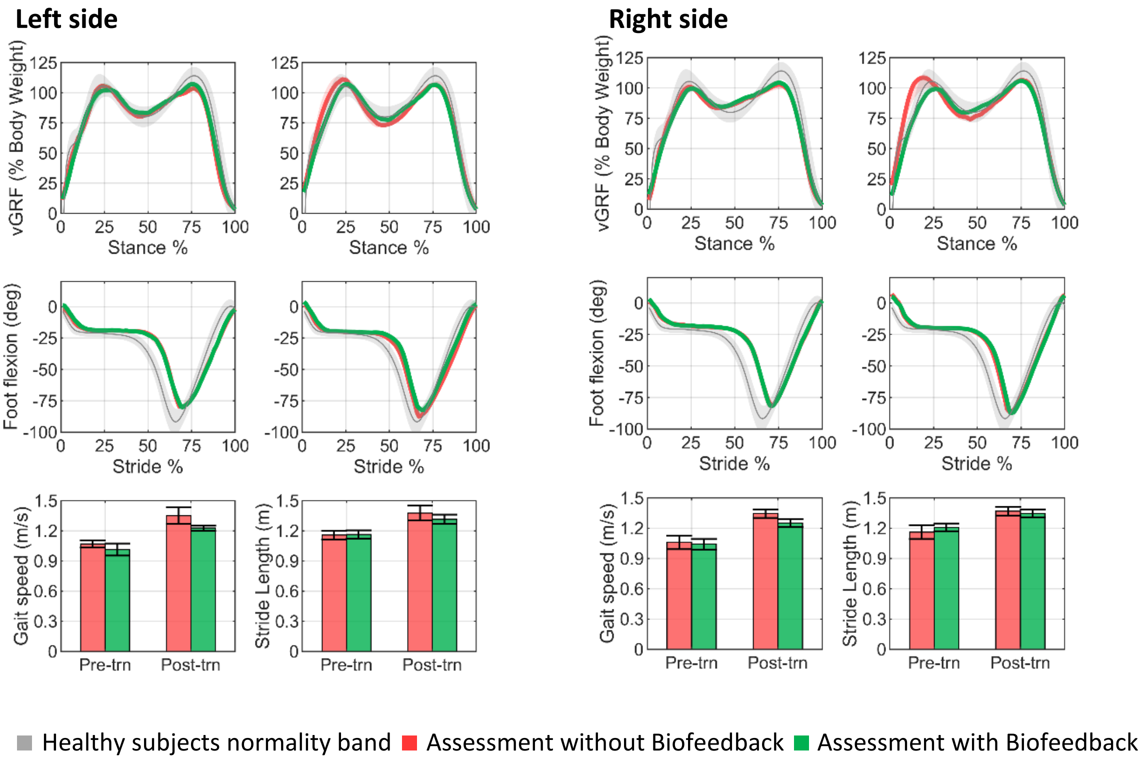

Subject 6

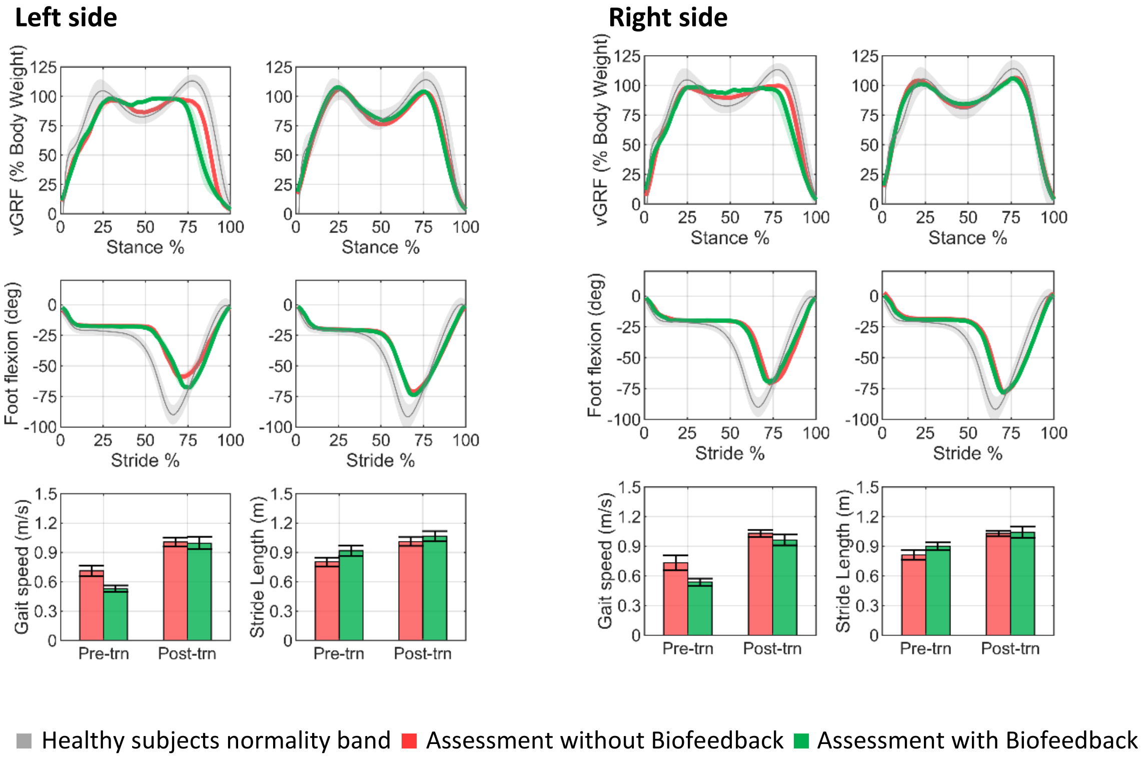

Subject 7

Left side **Right side**

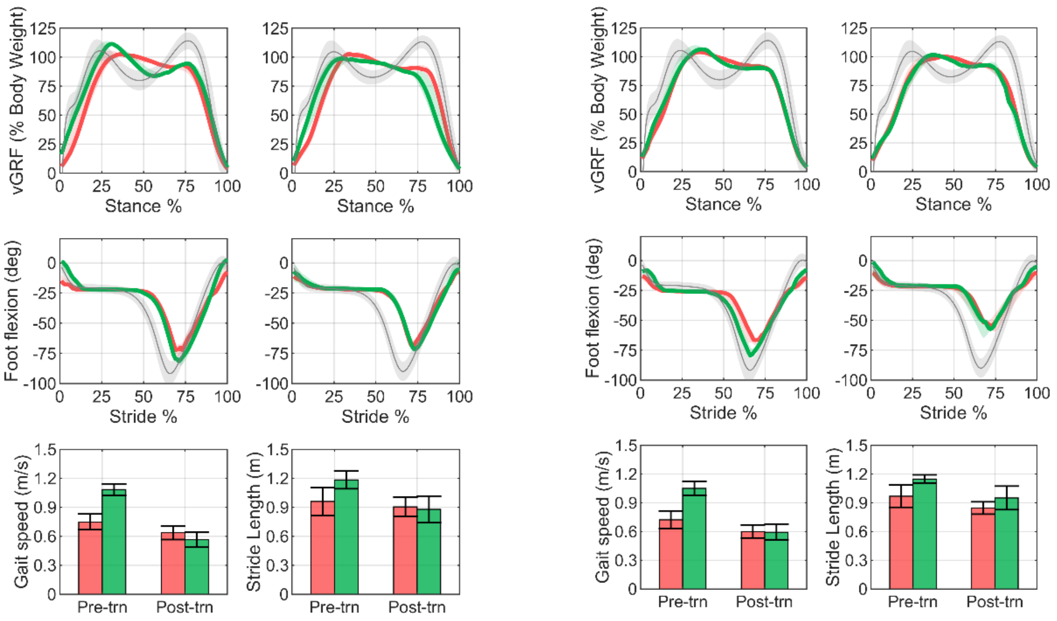

Healthy subjects normality band ■ Assessment without Biofeedback ■ Assessment with Biofeedback
